# The Multifaceted Responses of Plants to Visible and Ultraviolet Radiation

**DOI:** 10.3390/plants13050572

**Published:** 2024-02-20

**Authors:** Marco Santin, Antonella Castagna

**Affiliations:** 1 Department of Agriculture, Food and Environment, University of Pisa, via del Borghetto 80, 56124 Pisa, Italy; 2 Interdepartmental Research Center “Nutraceuticals and Food for Health”, University of Pisa, via del Borghetto 80, 56124 Pisa, Italy

## 1. Background

Plant organisms rely on light energy to drive the photosynthetic processes needed for their growth and development, inducing modifications at physiological, biochemical, and molecular levels. Therefore, throughout the centuries, they have tuned a multilevel network of responses from primary and secondary metabolisms to maximize the light harvesting reactions to minimize the cellular and molecular impairments that are likely to occur when there is excess light energy. In addition, several studies have demonstrated that specific wavelengths are able to trigger distinct physiological, biochemical, and molecular mechanisms, leading to specific plant responses that are strictly species- and cultivar-dependent. Therefore, in recent years, researchers have focused their attention on modulating the electromagnetic spectrum, particularly in terms of visible and ultraviolet (UV) wavelengths, in order to obtain desirable effects on the exposed plants, e.g., a higher plant productivity, an increased resistance to pathogens, and a higher content of health-promoting compounds. A few recent studies have also shown that the light-triggered intracellular signals can induce biochemical and molecular modifications in plant tissues and organs not directly exposed to that specific radiation. However, since the plant responses to variations in light quality and quantity are highly variable depending on both the irradiation conditions and the plant genotype, there is still much not known about the mechanisms behind these different plant responses. 

In line with this, the twelve manuscripts collected in this Special Issue of *Plants* cover many aspects related to the influence of irradiation, particularly UV wavelengths, on plant and microalgae organisms.

## 2. Main Findings Considered in this Special Issue

### 2.1. Transcriptomic Analysis of the Peel of UV-B-Exposed Peach Fruit Reveals Upregulation in Phenolic- and UVR8-Related Pathways [1]

UV-B has been observed to deeply influence plant secondary metabolism notably in post-harvest fruits. Thus, it is possible to increase their health-promoting phytochemical content by modulating the light quality and quantity. In this work, the authors adopted an omics-based approach to comprehensively investigate the molecular mechanisms triggered by exposing post-harvest peach (*Prunus persica*, cv. Fairtime) fruit to UV-B. The authors reported high activation of the UV-B- and phenylpropanoid-related pathways, which highly correlated with the strong increase in several phenolic compound subclasses (e.g., anthocyanins and dihydroflavonols) observed in previous studies from the same authors [2,3,4,5].

### 2.2. Effects of UV-B Radiation on the Performance, Antioxidant Response, and Protective Compounds of Hazelnut Pollen [6]

This paper deepens the knowledge of the effect of different UV-B irradiations, in terms of the distance from lamps and the exposure time, on hazelnut (*Corylus avellana*) pollen. The authors reported that, at the UV-B conditions tested, the treatments induced several stress-related responses, e.g., a decrease in the pollen viability, germination rate, and tube length, accompanied by a negative impact on the concentration of antioxidant compounds, e.g., total phenolics and flavonoids.

### 2.3. Hormonal Regulation in Different Varieties of Chenopodium quinoa Willd. Exposed to Short Acute UV-B Irradiation [7]

In this work, the researchers investigated the impact of two short acute UV-B exposures, 9.12 kJ m^−2^ d^−1^ and 18.24 kJ m^−2^ d^−1^, on the hormone biosynthesis and some photosynthetic and biochemical parameters in different varieties of quinoa (*Chenopodium quinoa* Willd.). The authors found marked differences between the cultivars tested, linking the increase in salicylic acid (SA) and jasmonic acid (JA) to a higher UV-B acclimation process, while correlating the changes in abscisic acid (ABA) content to the onset of a UV-B stress condition. Interestingly, they pointed out that both the parental lines and the breeding period strongly influenced the response to UV-B, and the degree of tolerance to UV-B radiation was not necessarily correlated with the more severe environmental conditions found in their natural habitat.

### 2.4. Foliar and Root Comparative Metabolomics and Phenolic Profiling of Micro-Tom Tomato (Solanum lycopersicum L.) Plants Associated with Gene Expression Analysis in Response to Short Daily UV Treatments [8]

Tomato (*Solanum lycopersicum* L.) is renowned globally for its significant value as a crop, offering both commercial profitability and nutritional advantages. Through an untargeted metabolomic analysis, the authors were able to identify secondary metabolites that were differentially modulated in leaves and roots of tomato plants exposed to an 11-day UV exposure (15 min/day, 1.19 kJ m^−2^ daily unweighted UV dose). An extensive modulation of foliar metabolome was found, especially concerning the phenylpropanoid pathway. Interestingly, the root metabolome was also affected by the UV exposure, even if this organ was not directly irradiated, suggesting that shoot-to-root signaling mechanisms occurred. However, while the phenolic compounds were reduced in UV-treated leaves, the roots displayed the opposite trend, suggesting that changes in nutrient uptake and root architecture also occur. Molecular data on UV-RESISTANCE LOCUS 8 (UVR8)-related and phenylpropanoid pathways confirmed the biochemical observations.

### 2.5. Growth and Biosynthesis of Phenolic Compounds of Canola (Brassica napus L.) in Response to Different Ultraviolet (UV)-B Wavelengths in a Plant Factory with Artificial Light [9]

This study sheds light on the effects of different UV-B doses on several morphological, physiological, and biochemical parameters of canola (*Brassica napus* L.) plants. The authors tested both short (280–300 nm) and long (300–320 nm) wavelengths. As expected, the higher and longer the UV-B doses, the more pronounced the decrease in plant growth, due to the short, thus more energetic and potentially harmful, UV-B wavelengths. In parallel, they also found a significant increase in the content of bioactive health-promoting compounds, underlining the importance of wisely choosing the best UV-B intensities to maximize the compound production without impairing the plant productivity.

### 2.6. Effect of UV-B Radiation on Flavonoid and Phenol Accumulation in Tempisque (Sideroxylon capiri Pittier) Callus [10]

In this work, the authors investigated UV-B’s potential to enhance the phenolic and flavonoid content of tempisque (*Sideroxylon capiri* Pittier) callus, as well as its effects on the morphology and growth. The results of the tests with four UV-B doses (0, 1, 2, 3, 4 h/day) showed that the content of both total phenolics and flavonoids increased, particularly after 4 weeks of treatment in the 4 h UV-B-treated group. In detail and aligned with the observations above, the authors detected a higher content of kaempferol, quercetin, and gallic acid after UV-B irradiation. Moreover, an enhanced growth rate was found in the UV-B-exposed callus. Since tempisque is considered a threatened species in Mexico, and in light of the high content of health-promoting compounds in its leaves and fruits, this study undoubtedly paves the way for the establishment of an in vitro propagation protocol of tempisque in order to contribute to its reforestation and exploitation for pharmaceutical and food industry purposes.

### 2.7. The Function of Flavonoids in the Diurnal Rhythm under Rapidly Changing UV Conditions—A Model Study on Okra [11]

This study investigated the impact of UV exposure on plant responses, particularly focusing on the role of flavonoids in UV absorption and changes in their amount according to the conditions of UV irradiation. Okra (*Abelmoschus esculentus*) plants were exposed to varying levels of UV radiation, revealing a UV-driven diurnal rhythm with a significant (up to 50%) increase in flavonoid content. In addition, the authors found that transferring plants from −UV to +UV conditions at 9:00 CDT triggered the start of the diurnal rhythm, while later transfers resulted in a delayed onset of flavonoid production, which only started the following day. Marked differences were clearly observed after a seven-day adaptation. Broadly, this mechanistic study on okra, underscores the significant influence of UV radiation on plant diurnal rhythms, revealing a relatively quick adaptation to changing UV conditions driven by flavonoid compounds.

### 2.8. To What Extent Are the Effects of UV Radiation on Grapes Conserved in the Resulting Wines? [12]

Although some studies have investigated the impact of UV-A and UV-B radiation on grape quality and metabolism, very little is known on the biochemical and organoleptic changes in the resulting wine. In this study, the authors found that the wine derived from the UV-exposed Tempranillo grapes had a higher content of flavonols (particularly quercetin-, kaempferol-, isorhamnetin-, and myricetin-glycosides) and hydroxycinnamic acids, contributing to a greater overall nutraceutical quality. Considering the volatile organic compounds (VOCs) analyzed, the wine resulting from the UV-B exposed grapes showed an increased production of esters, likely causing stronger fruity and therefore pleasant aromas. This result deserves deeper investigation since the released esters are produced only during the winemaking process. In conclusion, this work sheds light on the possibility of exploiting pre-harvest UV radiation to obtain desirable characteristics in the derived wine.

### 2.9. Narrowband 311 nm Ultraviolet-B Radiation Evokes Different Antioxidant Responses Than Broadband Ultraviolet [13]

In this study, the authors investigated the impact of UV-B supplementation (4 h/day, 4 days, narrowband 311 nm wavelength peak) on the enzymatic and the non-enzymatic antioxidant machinery of tobacco (*Nicotiana tabacum* cv. Petit Havana) leaves. The authors found that the irradiation increased the superoxide dismutase (SOD) activity but decreased the phenolic peroxidase activity. In addition, the H_2_O_2_ content was higher when plants were exposed to UV-B doses of 2.9–6 µmol m-2 s^−1^, while stronger UV-B doses determined a greater non-enzymatic antioxidant capacity, a higher SOD activity, and therefore a decreased H_2_O_2_ content. Interestingly, the plant response to the irradiation with narrowband (311 nm) UV-B lamps was partially different from the effects observed when the irradiation was conducted with broadband (313 nm) UV-B lamps [14,15].

### 2.10. Effects of Nocturnal UV-B Irradiation on the Growth, Flowering, and Phytochemical Concentration in Leaves of Greenhouse-Grown Red Perilla [16]

This work showed that nocturnal UV-B radiation, in the proper conditions, could positively influence the content of rosmarinic acid in red perilla (*Perilla frutescens*) without yield penalty, modifying the leaf phenotype. Indeed, the experimental design of the study comprised different exposure times (3 or 6 h/daily), different UV-B irradiances (0.05–0.2 W m^−2^), and different irradiation modes (continuously or intermittently). The authors found a worse leaf phenotypic quality, e.g., in plants irradiated intermittently with UV-B (0.1 W m^−2^) for 2 h and continuously for 3 h (0.1 W m^−2^) and 6 h (both 0.1 and 0.2 W m^−2^). Indeed, the leaves appeared smaller and greener compared to the controls, indicating a negative effect of UV-B radiation on anthocyanin accumulation. Therefore, the study suggested that is necessary to wisely choose the proper nocturnal UV-B conditions in order to obtain high-quality red perilla plants without physiological and biochemical impairments.

### 2.11. Anything New under the Sun? An Update on the Modulation of Bioactive Compounds Using Different Wavelengths in Agricultural Plants [17]

This comprehensive review deepens the knowledge of the effects, particularly on plant secondary metabolism, of different light conditions in several plants of nutritional interest in the pre-and post-harvest periods. After a brief introduction to the light perception and signal transduction mechanisms carried out by the different plant photoreceptors and a brief description of the main classes of health-promoting phytochemicals, the authors collected the more recent studies in the field, organizing the text according to the different light qualities. The authors evaluated how red and far-red light, green light, blue light, and UV-A and UV-B radiations influenced plant responses. They analyzed the complexity and variability of these responses across different plant species and cultivars, taking into account factors such as the irradiation intensity, duration of exposure, specific phytochemicals involved, and surrounding environmental conditions for each wavelength interval studied. Therefore, although modulating the light conditions might represent a promising eco-friendly way to increase the nutraceutical quality of edible plants, the authors underlined the need to deepen the study of this research topic.

### 2.12. The UV-B Irradiation Effect on Microalgae Performance in the Remediation of Effluent Derived from the Cigarette Butt Cleaning Process [18] 

Cigarette butts are among the most common sorts of waste, able to impair the vitality of several terrestrial and aquatic organisms due to their toxic compounds, e.g., heavy metals, polycyclic aromatic hydrocarbons, and aromatic amines. A sustainable way to recycle them is to incorporate them into soilless substrates, after a deep cleaning process. The contaminants in the resulting wastewater can be further neutralized through treatment with microalgae, to remove the aforementioned potentially harmful pollutants. In this article, the authors found that exposing different *Chlorella sorokiniana* strains to UV-B resulted in enhancing the non-enzymatic antioxidant machinery without negatively impacting the chlorophyll and carotenoid content. In addition, one strain tested showed a cross-resistance response induced by the UV-B towards the toxic pollutants in the cigarette butt wastewater, increasing the catabolic processes behind their neutralization.

## 3. Conclusions

This Special Issue comprehensively explores the biochemical and physiological responses exhibited by photosynthetic organisms, primarily focusing on agricultural plants, when subjected to the modulation of the electromagnetic spectrum. The insights gained from these investigations not only contribute to an enhanced understanding of the intricate mechanisms governing plant responses to different light conditions but also carry significant implications for diverse sectors, including the pharmaceutical and food industries. By broadening our knowledge in this domain, the Special Issue aims to uncover novel applications and potential advancements that may extend beyond conventional agricultural contexts, opening avenues for innovation and discovery in fields vital to both human health and nutrition.

We would like to thank the authors for their significant contributions to the topic in this Special Issue, as well as the MDPI team for their assistance during the editorial process.
